# 2479. Impact of the COVID-19 pandemic on the hospital acquisition of multidrug-resistant organism in two Greek hospitals

**DOI:** 10.1093/ofid/ofad500.2097

**Published:** 2023-11-27

**Authors:** Polyxeni Karakosta, Elisavet Kousouli, Sophia Vourli, Panagiota Christina Georgiou, Vasiliki Mamali, Olympia Zarkotou, Spyros Pournaras

**Affiliations:** Attikon University General Hospital, Medical School, National and Kapodistrian University of Athens, Athens, Greece, Athens, Attiki, Greece; Tzaneio General Hospital of Piraeus, Athens, Greece., Piraeus, Attiki, Greece; National and Kapodistrian University of Athens, Athens, Zakinthos, Greece; Attikon University General Hospital, Medical School, National and Kapodistrian University of Athens, Athens, Greece, Athens, Attiki, Greece; Tzaneio General Hospital of Piraeus, Piraeus, Attiki, Greece; Tzaneio General Hospital of Piraeus, Piraeus, Attiki, Greece; Attikon University General Hospital, Medical School, National and Kapodistrian University of Athens, Athens, Greece, Athens, Attiki, Greece

## Abstract

**Background:**

COVID-19 pandemic pushed back progress for combating antimicrobial resistance (AR). We aimed to provide a proxy measure for MDRO Hospital Acquisition in two Hospitals following COVID-19 pandemic.

**Methods:**

Active surveillance for MDROs recovered from any specimen collected >3 days post-admission, using laboratory data without patient’s clinical evaluation was conducted from Jan 2019 to Dec 2022 in Attikon & Tzaneio hospitals. MDROs included carbapenem-resistant (CR) *Enterobacteriaceae* (CRE), CR *Acinetobacter baumannii* (CRAB), CR *Pseudomonas aeruginosa* (CRPA), vancomycin-resistant *enterococci* (VRE) and methicillin-resistant *Staphylococcus aureus* (MRSA). Adult inpatients hospitalized in ICUs or non-ICU wards participated in the study. Surveillance definitions of MDRO Laboratory-Identified (LabID) Event and MDRO Infection/Colonization Incidence Density Rate (IDR) were based on the CDC MDRO/CDI Module criteria (January 2023). Patient days and susceptibility data of isolated CRE were collected monthly. Incidence rate ratio (95% CI) was calculated to examine changes in rates from 2019 (pre-pandemic) to 2020, 2021 and 2022 (pandemic).

**Results:**

During four years, 3,241 hospital-onset LabID Events, with no documented prior evidence of infection/colonization with the specific organism type, were recorded. The Infection/Colonization IDR for non-ICUs and ICUs was 0.59 and 6.69 per 1,000 patient days for CRE, 0.68 and 10.08 for CRAB, 0.19 and 1.41 for CRPA, 0.39 and 1.77 for VRE and 0.20 and 0.39 for MRSA, respectively. Compared to 2019, the CRE Infection/Colonization IDR raised significantly by 52% in 2020, 110% in 2021 and 146% in 2022 **only in non-ICUs;** non-significant increase was observed in ICUs. CRAB Infection/Colonization IDR increased constantly in non-ICUs after COVID-19 pandemic, but only during 2021 in ICUs. In CRE, KPC was the commoner carbapenemase (54%, 2019; 53%, 2020; 60%, 2021; 63%, 2022). CRPA infection/colonization significantly decreased during 2021 in ICUs (60%) but increased in 2022 in non-ICUs; VRE increased in 2022 in non-ICUs (61%) and MRSA remained unaltered.

**Figure:**
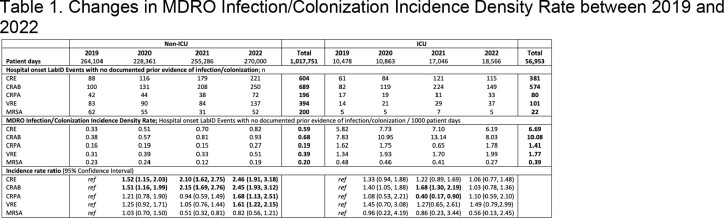

**Conclusion:**

MDRO Infection/Colonization IDR has further deteriorated during the pandemic with CRE and CRAB having leading role.

**Disclosures:**

**All Authors**: No reported disclosures

